# A review of the fernane-type triterpenoids as anti-fungal drugs

**DOI:** 10.3389/fphar.2024.1447450

**Published:** 2024-08-21

**Authors:** Chun-Yue Liu, Lu Zhang, Si-Xuan Liu, Yong-Fu Lu, Chang Li, Yue-Hu Pei

**Affiliations:** ^1^ Department of Medicinal Chemistry and Natural Medicine Chemistry, College of Pharmacy, Harbin Medical University, Harbin, China; ^2^ Department of Chemistry, College of Pharmacy, Harbin Medical University, Daqing, China

**Keywords:** triterpenoid, enfumafungin, ibrexafungerp, anti-fungal, fernane type

## Abstract

Human fungal pathogens could cause a broad plethora of infections in both the immunocompetent and immunocompromised host. Fungal infections have become important causes of morbidity and mortality in recent years, the current arsenal of anti-fungal therapies was restricted. Ibrexafungerp was a novel, highly bioavailable glucan synthase inhibitor formulated for both intravenous and oral administration being developed by Scynexis; it was also the first novel anti-fungal drug class approved in more than 20 years. Ibrexafungerp was one semi-synthetic derivative of enfumafungin, a natural product isolated from fungi. This review reported the discovery of enfumafungin and ibrexafungerp, their anti-fungal mechanism, summed up 63 fernane-type triterpenoids from natural products, including 49 from plants, 9 from fungi and 5 from lichen. In addition, the review summarized the progress of enzymes responsible for the biosynthesis of type II fernane triterpenoid (enfumafungin skeleton) and type I fernane triterpenoid (polytolypin skeleton). The good example kept our confidence up for searching for new leading compounds and discovering drugs from fungi.

## Introduction

Human fungal pathogens could cause a broad plethora of infections in both the immunocompetent and immunocompromised host. The incidence of fungal infections has increased significantly over the last several decades (Yu et al., 2021; Li Z. et al., 2023). Consequently, fungal infections have become important causes of morbidity and mortality (Gamal et al., 2021). The most common clinical pathogenic fungi were *Candida albicans*, *Aspergillus fumigatus* and *Cryptococcus neoformans* (Liu et al., 2023). Among them, *C. albicans* was an opportunistic pathogen, which could cause superficial infection and lead to mortal systemic infections, especially in immunocompromised patients (Gamal et al., 2021). It was reported that up to 70%–75% of women experienced an episode of Vulvovaginal candidiasis (VVC) and it has been estimated that approximately 10%–15% of asymptomatic women were colonized with a *Candida* species (Anderson et al., 2004; Sobel, 2007).

The current arsenal of anti-fungal therapies was restricted, with only three major drug classes available as first-line therapies (Armstrong-James, 2022). The first ones were the polyenes. They had very broad spectrum anti-fungal activity, but their clinical use was often limited due to their nephrotoxicity caused by non-specific binding to mammalian sterols (Armstrong-James et al., 2020); The second ones were azole derivatives. They were better tolerated than the polyenes and had the advantage of being orally bio-available; However, inhibition of host P450 enzymes resulted in a plethora of drug-drug interactions (DDIs) that present challenges for patients taking multiple medications (Nwankwo et al., 2022); The third ones were the echnocandins, which were the most recent class of anti-fungals, compromised the integrity of the fungal cell wall, eventually leading to cell lysis under osmotic stress, through the inhibition of *β*-1,3-glucan synthase (GS), which produced *β*-1,3-glucan, a critical component of the cell wall. Due to the specificity of their target for fungal cells, the echinocandins have demonstrated improved tolerability and fewer DDIs compared to the polyenes and azoles. The echinocandins were now recommended as first line therapy for invasive candidiasis, as a result of these advantages and because of their broad spectrum of activity for *Candida* species. Restriction to parenteral administration due to poor oral bioavailability constituted a significant limitation for echinocandin therapy (Perlin, 2011). Besides, significant resistance of *Candida glabrata* and *Candida auris* to echinocandins has emerged (Armstrong-James, 2022). Therefore, developing new drugs and strategies to combat fungal infections was crucial.

Fortunately, ibrexafungerp (formerly named SCY-078) was a novel, highly bio-available glucan synthase inhibitor formulated for both intravenous and oral administration currently being developed by Scynexis (Jersey City, NJ, United States). It has been approved on June 2021 in the United States for the treatment of vulvovaginal candidiasis (VVC), and it was the first novel anti-fungal drug class approved in the past 20 years (Azie et al., 2020; Waterer, 2021; Angulo et al., 2022; Sucher et al., 2022). Ibrexafungerp exerted concentration-dependent fungicidal activity against *Candida* species and retained good *in vitro* activity against most echinocandin-resistant strains and fluconazole-resistant strains (Nunnally et al., 2019; Davis et al., 2020; Gamal et al., 2021; Mesquida et al., 2021; Sobel et al., 2021; Wiederhold et al., 2021). In addition, Ibrexafungerp has been shown to be safe and effective in the treatment of vulvovaginal candidiasis caused by *C. albicans* in phase II and phase III clinical trials (Vazquez et al., 2021; Daraskevicius et al., 2022; Schwebke et al., 2022; Sobel et al., 2022; Goje et al., 2023). It was approved for vulvovaginal candidiasis in adult and postmenarchal pediatric females and was given as two 150-mg tablets orally, administered 12 h apart (Sucher et al., 2022). Clinical trials were ongoing for recurrent and complicated vulvovaginal candidiasis as well as invasive candidiasis and pulmonary aspergillosis (Colombo and Vazquez, 2021; Phillips et al., 2022; Sucher et al., 2022).

**FIGURE 1:**
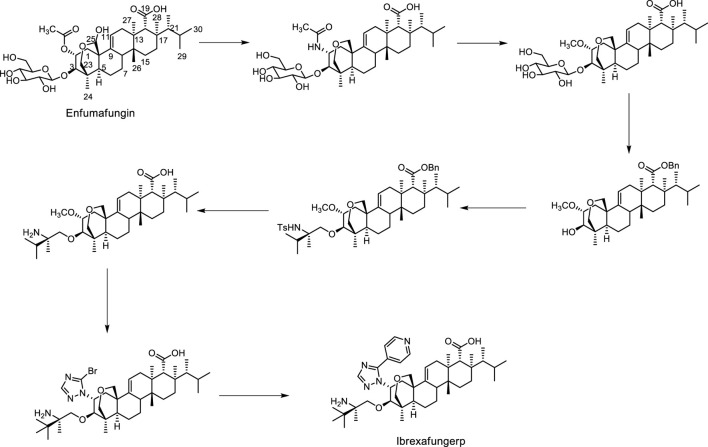
The synthetic procedure from enfumafungin to ibrexafungerp.

Structurally, ibrexafungerp was one semi-synthetic derivative originated from fernane-type triterpenoid glycoside enfumafungin (Figure 1), which was firstly isolated from an endophytic fungus in 2000 (Peláez et al., 2000; Apgar et al., 2021; Lee, 2021). Thus, herein we summarized the discovery procedure of ibrexafungerp from enfumafungin, the modified derivatives of enfumafungin, the mechanism of them, as well as the fernane type triterpenoids isolated from natural products up to now, and the biosynthesis clues of this kind of triterpenoids.

## The discovery procedure, from enfumafungin to ibrexafungerp

Enfumafungin was a fernane-type triterpene glycoside that was originally isolated from the fermentation of *Hormonema* sp. by Merck in 2000 (Peláez et al., 2000; Schwartz et al., 2000; Hector and Bierer, 2011). Then it was found out that the natural product inhibited the synthesis of 1,3-*β*-*D*-glucan, a necessary component of the fungal cell wall, by inhibiting the enzyme glucan synthase (Onishi et al., 2000). Unfortunately, it showed weak activity in a murine model of disseminated candidiasis due to limited stability *in vivo*. Therefore, since its isolation, Merck undertook the challenge of improving oral efficacy and pharmacokinetic properties of enfumafungin through semi-synthetic modification of the natural product. Firstly, enfumafungin was elaborated by a series of chemical transformations such as reduction of the hemiacetal, protection of the carboxylic acid functionality at C-19 as a benzyl ester and deglycosylation to produce several key intermediates, shown in Figure 1, from which most semi-synthetic derivatives reported in the patent were prepared. Then, In 2010, the patent WO2010019204 described 318 examples of 25-deoxyenfumafungins, with a substituted triazole group or 1,2,3-triazole, pyrazole, imidazole at C-2, and an amino-containing alkoxy group at C-3 (Merck and Co, 2010). The triazole ring of the exemplified compounds often contained an aryl or heteroaryl substituent. Biological activity for many of the derivatives against *C. albicans* were reported, with nine of the exemplified compounds having reported activities <1 ng/mL. One of these compounds was MK-3118 (the formerly name of ibrexafungerp), an orally active enfumafungin derivative, which was example 173 in the application (Liberator et al., 2010; Merck and Co, 2010). Thus, MK-3118 has been evaluated using various *in vitro* and *in vivo* assays. In a glucan synthase assay, MK-3118 resulted in an half maximal inhibitory concentration (IC_50_) of 0.6 ng/mL for partially purified microsomal enzyme derived from *C. albicans*, an improvement of three orders of magnitude compared with the 25-deoxyenfumafungin parent compound (Balkovej et al., 2009; Hector and Bierer, 2011). Using glucan synthase prepared from *A. fumigatus*, an IC_50_ of 0.0017 μg/mL was determined for MK-3118, compared with 0.5 ng/mL for caspofungin (Motyl et al., 2010; Hector and Bierer, 2011). This compound showed minimum inhibitory concentration (MIC) values of ≤1 μg/mL and ≤0.015 μg/mL against 160 strains of 7 *Candida* spp. and 40 *Aspergillus* spp., respectively. Moreover, MK-3118 has been evaluated in standard rodent models of candidiasis and aspergillosis. Following administration to mice, the half-life of MK-3118 was determined to be 4.4 h, with 34% oral bioavailability. Overall, the preclinical results demonstrate a comparable level of activity for MK-3118 against *Candida* spp. compared with the echinocandin caspofungin, while mouse efficacy results for aspergillosis suggested a somewhat inferior response compared with caspofungin (Hector and Bierer, 2011; Jiménez-Ortigosa et al., 2013; Pfaller et al., 2013a; Pfaller et al., 2013b).

**FIGURE 2:**
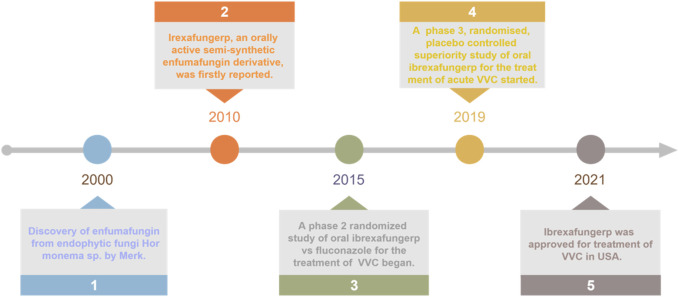
Key milestones in the development of oral ibrexafungerp from enfumafungin.

Since 2015, several clinical trials have been conducted to determine the optimal dosing regimen and to assess the efficacy and safety of ibrexafungerp in the treatment of acute VVC, including phase II clinical trial SCY-078-203 (Nyirjesy et al., 2021; Sucher et al., 2022), and phase III clinical trial VANISH 303 and VANISH 306 (Sobel et al., 2022; Sucher et al., 2022). As expected, ibrexafungerp was approved by Food and Drug Administration (FDA) for the treatment of acute VVC in 2021 (Figure 2). It was currently the only oral non-azole agent approved for the treatment of VVC, it was also the first drug in a new class of anti-fungal agents known as fernane-type triterpenoids. In addition, ibrexafungerp was being studied for the treatment of invasive candidiasis and aspergillosis in ongoing clinical trials, and was approved for recurrent VVC (RVCC) in December 2022 (Sucher et al., 2022; Phillips et al., 2023).

## The mechanism of ibrexafungerp and enfumafungin

In 2000, Onishi group reported the identification of four triterpenoids as anti-fungal agents. One of the four compounds, enfumafungin, showed significant bioactivity, which could be comparable to that of the control L-733560 (Onishi et al., 2000). Moreover, they also found that enfumafungin specifically inhibited glucan synthesis in whole cells and in (1,3)-*β*-*D*-glucan synthase assays, altered the morphologies of yeasts and molds. To further explore the effects of enfumafungin on glucan synthase, enfumafungin was tested against *Saccharomyces cerevisiae* strains with point mutations in FKS1 (Zhao et al., 2023), the gene encoding the vegetatively expressed large subunit of glucan synthase. The result indicated that enfumafungin produced a unique response in *S. cerevisiae* strains with point mutations in FKS1, which support the conclusion that enfumafungin was specific inhibitors of glucan synthase. Thus, enfumafungin represented a new group of (1,3)-*β*-*D*-glucan synthase inhibitors (Douglas et al., 1997; Onishi et al., 2000). Since ibrexafungerp was reported in 2010, it has been evaluated using various *in vitro* and *in vivo* assays, its mechanism was also discussed (Hector and Bierer, 2011; Jiménez-Ortigosa et al., 2013; Pfaller et al., 2017). It was the first triterpenoid of (1, 3)-*β*-*D*-glucan inhibitor with good oral bioavailability (Hector and Bierer, 2011).

## Fernane-type triterpenoids from natural products

Fernane-type triterpenoids were a rare type triterpenoids, with unique 6/6/6/6/5 skeleton. They have been isolated from plants, fungi and bacteria. It was unusual migrated hopane triterpenoids. A number of fernane-type triterpenoids were isolated and identified since 1980s. Several of them showed anti-fungal bio-activity.

**FIGURE 3:**
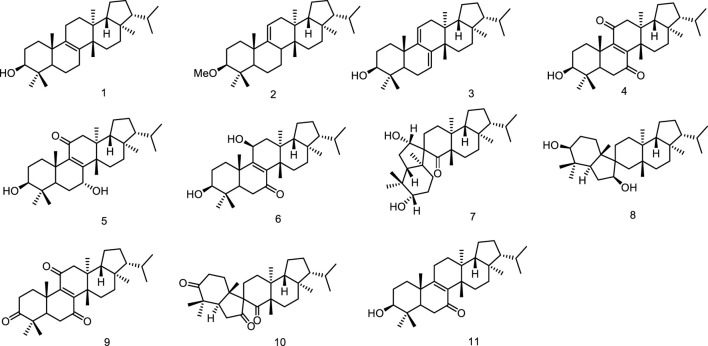
Fernane type tri-terpenoids (1–11) from natural products.

In the course of search for the biologically active constituents of *Euphorbia supina* Rafin, a toxic annual weed which was used as a folk medicine for the treatment of gastroenteric diseases and for healing suppurated swelling, Tanaka et al. found that the plant contained a number of triterpenoids bearing a novel and biogenetically interesting fernane skeleton, migrated fernane skeleton and seco-fernane skeleton. Their structures were established as fern-8-en-3β-ol (1), 3β-methoxy-fern-9 (11)-ene (2), ferna-7,9 (11)-dien-3β-ol (3), 3β-hydroxyfern-8-en-7,11-dione (4), 3β/7a-dihydroxyfern-8-en-1l-one (5), 3β/11β-dihydroxyfern-8-en-7-one (6), and spirosupinanonediol (7) (Tanaka and Shunyo, 1988; Tanaka and Shunyo, 1989) as well as neospirosupinanonediol (8), supinenolone D (9) and neospirosupinanetrione (10), supinenolone E (11) (Tanaka and Shunyo, 1991a; Tanaka and Shunyo, 1991b) (Figure 3).

**FIGURE 4:**
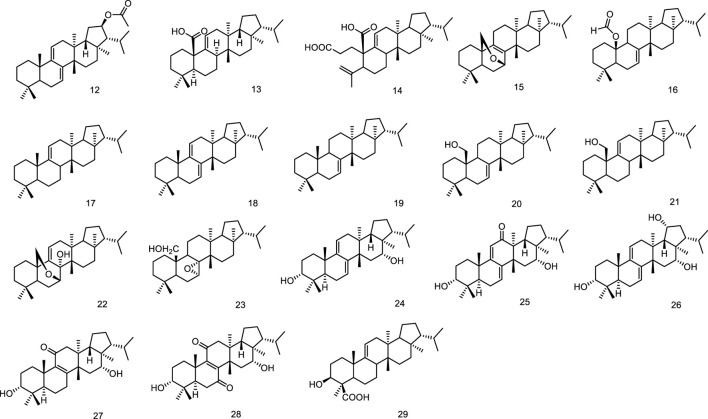
Fernane type tri-terpenoids (12–29) from natural products.

Phytochemical studies on *Sericostoma pauciflorum* provided a new fernane-type triterpenoid sericostinyl acetate (12) (Ayatollahi et al., 1991). A new fernane type triterpene has been isolated from the fern *Adiantum venustum*. Its structure has been elucidated to be fern-9 (1l)-en-25-oic acid (13) by Banerjee et al. (1991). A new seco-triterpene-dienoic acid was isolated from *Euphorbia Chamaesyce*, its structure was established as 3,4-seco-8βH-ferna-4 (23),9 (11)-dien-3-oic acid (14) (Tanaka et al., 1996). Ageta et al. isolated a series of fernane triterpenoids from Adiantum cuneatum, including 7β,25-epoxyfern-8-ene (15), 25-norfern-7-en-10β-yl formate (16), fern-9 (11)-ene (17), ferna-7,9 (11)-diene (18), fern-7-ene (19) (Shiojima et al., 1997a), fern-7-en-25-ol (20), fern-9 (11)-en-25-ol (21), 7β,25-epoxyfern-9 (11)-en-8α-ol (22), 7α,8α-epoxyfernan-25-ol (23) (Shiojima et al., 1997b). Five new fernane type triterpenoids with α hydroxy group at C-3, ferna-7, 9 (11)-diene-3α, 16α-diol (24), 3α 16α-dihydroxyferna-7, 9 (11)-dien-12-one (25), ferna-7, 9 (11)-diene-3α, 16α, 19α-triol (26), 3α, 16α-dihydroxyfern-8-en-11-one (27) and 3α, 16α-dihydroxyfern-8-ene-7, 11-dione (28), were isolated from the leaves of *Lonicera gracilipes* var. glandulosa MAXIM (Kikuchi et al., 1999). A new fernane-type triterpene, named integnfolin (29), was isolated and characterized from the aerial parts of *Teucrium integnfolium*, the structure of the new compound was deduced to be 3β-hydroxy-fern-9 (11)-en-23-oic acid through its spectral properties and X-ray crystallographic analysis (Chen et al., 2000) (Figure 4).

**FIGURE 5:**
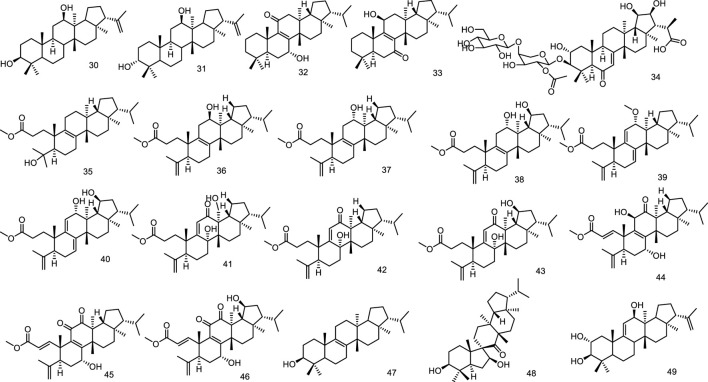
Fernane type tri-terpenoids (30–49) from natural products.

Two new fernane-type triterpenes, namely betufernanediol A (30) and betufernanediol B (isomers) (31) have been isolated from the stem bark of *Betula pendula* (Mukhtar et al., 2003). Two new fernane triterpenoids, 7α-hydroxyfern-8-en-11-one (32) and 11β-hydroxyfern-8-en-7-one (33), were isolated from the methyl alcohol extract of the leaves of *Angiopteris palmiformis* (Chen et al., 2010). The aerial parts of *Spergula fallax* afforded one new fernane class triterpenoid, 3-*O*-[*β*-D-glucopyranoside-(1→4)-*O*-*α*-L-(2-*O*-acetyl)-arabinopyranosyl]-2*α*,3*β*,19*β*,20*β*-tetrahydroxyfern-7en-6-oxo-29-oic acid (34) (Hamed et al., 2014). Belamcandaoids C-N (35–46), twelve new 3,4-seco-triterpenoids belonging to fernane type, were isolated from the seeds of *Belamcanda chinensis* (Song et al., 2021). Extensive phytochemical investigation on the whole herbs of *Euphorbia hypericifolia* led to the isolation of lots of structurally diverse triterpenoids, including two fernanes isomotiol (47) and teuviscin A (48) (Hu et al., 2021). One fernane-type triterpenoid, fern-9 (11)-ene-2α,3β-diol (49) was isolated from the stem bark of *Ancistrocarpus densispinosus* Oliv (Figure 5).

A thin-layer chromatography-biography screening guided phytochemical investigation for anti-fungal constituents from a lichen, *Lobaria kurokawae* Yoshim., led to the isolation of a pentacyclic triterpenoid, retigeric acid B (50). It exhibited anti-fungal activity alone against both azole-sensitive and -resistant *C. albicans* isolates. Furthermore, when it was combined with azoles, strong synergy was observed against azole-resistant strains, with synergistic or indifferent effects observed against azole-sensitive strains. 50 was an acid with anti-fungal activity that possibly has activity either in facilitating the uptake of azoles or in enhancing the membrane damage associated with the action of the azoles (Sun et al., 2009). Three novel fernane-type triterpenoids lobarialides A-C (51–53), together with two known ones retigeric acids A and B (54 and 50), were isolated by anti-fungal bioassay-guided from fractionation of the lichen Lobaria kurokawae. The isolated compounds were evaluated against the sensitive strain of the pathogenic fungus *Candida* albicans (CA2) according to the NCCLS method. 50 showed the best bioactivity with MIC values at 4.0 μg/mL. These results indicated that the anti-fungal activity of these compounds was closely related to the number of COO groups in the respective compounds. As the number of the COO groups increases, the activity is magnified. Although compound 50 exhibited weaker anti-fungal activity than these glycosides, it is the first anti-fungal fernane-type triterpenoid aglycone reported until 2009 (Wang et al., 2009).

**FIGURE 6:**
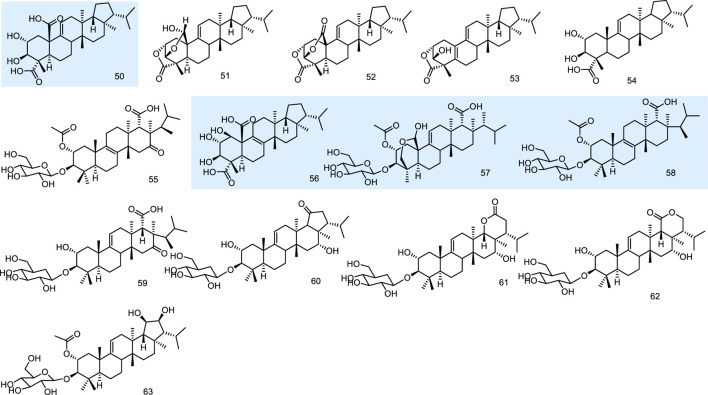
Fernane type tri-terpenoids (50–63) from natural products.

Fernane-type triterpenoids are relatively uncommon as fungal metabolites, while WF11605 (55) from the strain F11605, displayed anti-inflammatory effects (Shigematsu et al., 1992; Tsujii et al., 1992). Polytolypin (56), exhibiting anti-fungal and antibiotic activity, has been isolated from cultures of *Polytofypa bystricis* (JS189), a fungal colonist of porcupine dung. Polytolypin was found to be active against *C*. *albicans* (ATCC 14053), producing an 11-mm zone of inhibition in a standard disk assay at 80 pg/disk (Gamble et al., 1995). Enfumafungin (57) from *Hormonema* sp. (Schwartz et al., 2000) and fuscoatroside (58) from *Aspergillus flavussclerotia* (Joshi et al., 2002) displayed significant anti-fungal activities. Then four new triterpenoid glycosides, kolokosides A-D (59–62), were isolated from cultures of a Hawaiian wood-decay fungus (*Xylaria* sp.). 59 exhibited activity against Gram-positive bacteria (Deyrup et al., 2007). Peniciside (63), a new fernane triterpenoid glycoside, was isolated from the EtOAc extract of the solid-state fermented rice culture of the fungus *Penicillium* sp. 169. 63 was the first example of a fernane triterpenoid glycoside with two hydroxyls at C-19 and C-20 (Yuan et al., 2012) (Figure 6).

## Biosynthesis of enfumafungin

**FIGURE 7:**
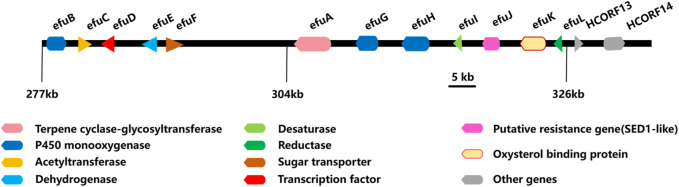
Biosynthesis gene cluster of enfumafungin.

The biosynthesis of enfumafungin was still unclear until Kuhnert et al. reported on the preliminary identification of the enfumafungin biosynthetic gene cluster (BGC) based on genome sequencing, phylogenetic reconstruction, gene disruption, and cDNA sequencing studies. It was interesting to find out that enfumafungin synthase (efuA) consisted of a terpene cyclase domain (TC) fused to a glycosyltransferase (GT) domain and thus represented a novel multifunctional enzyme (Figure 7). Moreover, the TC domain bore a phylogenetic relationship to bacterial squalene-hopene cyclases (SHC) and included a typical DXDD motif within the active center suggesting that efuA evolved from SHCs. Phylogenetic reconstruction of the GT domain indicated that this portion of the fusion gene originated from fungal sterol GTs (Kuhnert et al., 2018).

**FIGURE 8:**
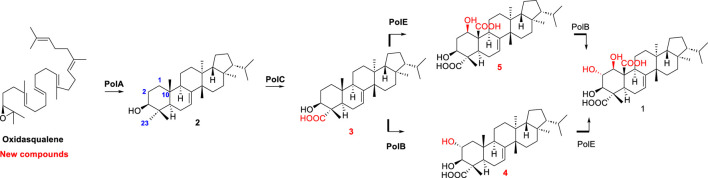
Biosynthesis pathway of polytolypin.

Then, in 2021, Li et al. reported the biosynthesis of group II fernane type triterpenoid, polytolypin. The authors have identified the biosynthetic gene cluster of polytolypin from its producer *Polytolypa hystricis* UAMH7299 and characterized its biosynthetic pathway. A new fernane-type triterpene cyclase was responsible for the biosynthesis of motiol, which was subsequently oxidized by three cytochrome P450s to give polytolypin. Moreover, the three P450 enzymes were employed in combination with two other new fungal fernane-type cyclases for isomotiol and fernenol biosynthesis to generate six polytolypin analogues (Li et al., 2023) (Figure 8).

## Discussion

Inventing and developing a new medicine is a long, complex, costly and highly risky process that has few peers in the commercial world. Research and development (R&D) for most of the medicines available today has required 12–24 years for a single new medicine, from starting a project to the launch of a drug product. In addition, many expensive, long-term research projects completely fail to produce a marketable medicine (Lombardino and Lowe, 2004). Fortunately, it took only 21 years from discovery of enfumafungin to use of ibrexafungerp as an anti-fungal drug approved by FDA.

Literature reviews demonstrated that natural products account for around 50% of approved new drugs in past few years (Newman and Giddings, 2013; Newman and Cragg, 2020). In recent years, since the discovery of penicillin, the first β-lactam antibiotic, fungi provided modern medicine with important antibiotics for curing life threatening infectious diseases, fungal secondary metabolites have revolutionized medicine yielding blockbuster drugs and drug leads of enormous therapeutic (Elander, 2003; Aly and Debbab, 2011). Ibrexafungerp, whose leading compound was enfumafungin, which was firstly isolated from fungus *Hormonema* sp., also represented one example of typical agents originated from fungi. In addition, it was still challenge for drug discovery from microorganism in recent years, especially using the classical bioactivity-guided isolation method to search for the leading compounds from natural products. With the development of genome sequencing, recent genome mining and biosynthetic engineering could enable faster and more efficient production of more leading compounds (Méndez et al., 2015; Hindra et al., 2017; Atanasov et al., 2021). Besides, co-cultivation using helper strains could also promote access to discovery of leading compounds (Abdel- Razek et al., 2018; Moussa et al., 2019).

Triterpenoids have been clinically used widely, such as fusidic acid, ginsenoside Rg3 et al. Among them, fusidic acid, which was used for treatment of skin infections caused by *Staphylococcus aureus*, was a group of fungi-derived 29-nor protostane triterpenoid antibiotics, targeting elongation factor G to inhibit bacterial protein synthesis (Cao et al., 2020; Chavez et al., 2021). Similarly, enfumafungin, represented the first fernane type-triterpenoid with anti-fungal activity, blocking the synthesis of the fungal cell wall polymer *β*-(1,3)-*D*-glucan, whose derivative ibrexafungerp received FDA for the treatment of Vulvovaginal candidiasis (VVC) in June 2021, and was subsequently approved for recurrent VVC (RVCC) in December 2022. Fluconazole is the only oral medication FDA approved for VVC before. None of these treatments are FDA approved for RVVC. Ibrexafungerp, a triterpenoid fungicidal agent, was FDA approved in 2021, becoming the first oral non-azole agent for VVC (Phillips et al., 2023). Ibrexafungerp provides an alternative oral option for treatment of acute, severe VVC. It is the only FDA approved anti-fungal for RVVC. Currently, the population could benefit from this drug are those with azole allergy, non-albicans or azole resistant albicans species, or other azole contraindications such as drug interactions (like statins or tricyclics) (Phillips et al., 2022; Phillips et al., 2023).

In conclusion, from enfumafungin, a metabolite from fungi, to ibrexafungerp, one “first-in-class” anti-fungal agent. It took 21 years from discovery of enfumafungin to use of ibrexafungerp as an marketable anti-fungal medicine approved by FDA, during this period, a number of derivatives and similar type natural products were synthesized or isolated, their bioactivity-structure relationship were discussed. The good example kept our confidence up for searching for new leading compounds and discovering drugs from fungi. Moreover, close attention has been paid to the biosynthetic pathway of fernane-type triterpenoids, it will become possible that ibrexafungerp be produced by bio-synthetic way soon.
